# DNA sequence diversity and the origin of cultivated safflower (*Carthamus tinctorius *L.; Asteraceae)

**DOI:** 10.1186/1471-2229-7-60

**Published:** 2007-11-06

**Authors:** Mark A Chapman, John M Burke

**Affiliations:** 1 Department of Plant Biology, Miller Plant Sciences Building, University of Georgia, Athens, GA 30602, USA

## Abstract

**Background:**

Safflower (*Carthamus tinctorius *L.) is a diploid oilseed crop whose origin is largely unknown. Safflower is widely believed to have been domesticated over 4,000 years ago somewhere in the Fertile Crescent. Previous hypotheses regarding the origin of safflower have focused primarily on two other species from sect. *Carthamus *– *C. oxyacanthus *and *C. palaestinus *– as the most likely progenitors, although some attention has been paid to a third species (*C. persicus*) as a possible candidate. Here, we describe the results of a phylogenetic analysis of the entire section using data from seven nuclear genes.

**Results:**

Single gene phylogenetic analyses indicated some reticulation or incomplete lineage sorting. However, the analysis of the combined dataset revealed a close relationship between safflower and *C. palaestinus*. In contrast, *C. oxyacanthus *and *C. persicus *appear to be more distantly related to safflower.

**Conclusion:**

Based on our results, we conclude that safflower is most likely derived from the wild species *Carthamus palaestinus*. As expected, safflower exhibits somewhat reduced nucleotide diversity as compared to its progenitor, consistent with the occurrence of a population genetic bottleneck during domestication. The results of this research set the stage for an investigation of the genetics of safflower domestication.

## Background

Safflower (*Carthamus tinctorius *L.) is a thistle-like, self-compatible, annual, diploid (2*n *= 24) herbaceous crop that thrives in hot, dry climates, and is capable of surviving on minimal surface moisture. It is believed to have been domesticated somewhere in the Fertile Crescent region over 4,000 years ago [1]. Following its initial domestication, safflower cultivation is thought to have expanded to both the east and west [2], with Knowles [3] ultimately recognizing seven "centers of similarity" (the Far East, India-Pakistan, the Middle East, Egypt, Sudan, Ethiopia and Europe). Safflower lines native to each 'center' are remarkably similar in height, branching, spines, flower color and head size; however, consistent morphological differences are maintained between the centers.

For centuries, safflower was grown on a local scale for its flowers, which served as a source of dye (carthamine) for textiles and food coloring, as well as for use in religious ceremonies [4]. Floral extracts were also used to flavor foods, and have historically been valued for their numerous medicinal properties. Cultivation of safflower in the New World commenced in 1899, and commercial production of safflower as an oilseed crop began in the 1950s [5]. More recently, there has been growing interest in safflower for its potential as a large-scale production platform for plant-made pharmaceuticals [6,7].

To date, phylogenetic investigations of *Carthamus *species have focused on either delimiting the sections within the genus [e.g. [8,9]], or on the development of DNA fingerprinting methodologies for investigating relationships amongst safflower cultivars [e.g. [10]]. Relationships between closely-related species within each section are, however, only poorly understood, and details surrounding the origin and early evolution of safflower are lacking. What we currently know is that safflower belongs to a group of closely related diploid species (sect. *Carthamus*; all 2*n *= 24 chromosomes [11]) whose ranges extend from central Turkey, Lebanon, and Israel in the west to northwestern India in the east. In addition to *C. tinctorius*, this section is composed of *C. curdicus *Hanelt, *C. gypsicola *Iljin, *C. oxyacanthus *Bieb. (= *C. oxyacantha *M. Bieb.), *C. palaestinus *Eig, and *C. persicus *Desf. ex Willd. (= *C. flavescens *Spreng) [8,12]. However, these species all exhibit some degree of cross-compatibility with one another [reviewed in [12,13]] and thus reproductive isolation alone cannot be used to delimit the species. *Carthamus curdicus *and *C. palaestinus *exhibit restricted geographical distributions (Northern Iran and Southern Israel, respectively), whereas *C. persicus*, *C. gypsicola *and *C. oxyacanthus *are more widely distributed throughout the Middle East [12].

Hypotheses regarding the origin of safflower have focused on *C. oxyacanthus *or *C. palaestinus *as the most likely progenitors, although *C. persicus *has also been suggested as a possible progenitor [14]. Here we report on levels of nucleotide diversity within and among species of sect. *Carthamus*, and investigate the origin of cultivated safflower using data derived from seven nuclear genes.

## Results

### DNA sequence diversity

Sequence data were collected from all seven gene regions for each of the 23 individuals surveyed (Tables 1 and 2), encompassing all species within sect. *Carthamus*. All sequences have been deposited in the Genbank Data Library and are available under accession nos. EF483951–EF483974, EF483983–EF484014, EF519712–EF519729, EF519732–EF519751, EF519754–EF519770, EF519774–EF519792, EF519795–EF519811, EF519815–EF519834 and EF519838–EF519857. Excluding indels, sequence lengths varied from 365 to 621 base pairs (bp) per locus, and all sequences included both exons and introns (Table 3). Thus, we were able to analyze 3239 bp of aligned sequence per individual with 1317 bp (40%) coming from exons and 1922 bp (60%) coming from introns. Across taxa, the number of indel polymorphisms per locus varied from four to thirteen, with a total of 53 indels in the data set. All indels were excluded from the analyses of nucleotide polymorphism.

**Table 1:** List of accessions surveyed, including information on the source of each sample.

Species	Code	Cultivar/Accession	PI/herbarium^a^	Origin^b^
*C. tinctorius *L.	saffW	W6 6730	PI 576995	China*
*C. tinctorius *L.	saffL	LESAF 494	PI 603207	Canada
*C. tinctorius *L.	saffE	ENANA	PI 610263	Spain*
*C. tinctorius *L.	saffU	USB	PI 560163	USA
*C. tinctorius *L.	saffAZ	ARIZ SAFF COMP III	PI 572418	USA
*C. tinctorius *L.	saffAC	AC SUNSET	PI 592391	Canada
*C. tinctorius *L.	saff1063	BJ-1063	PI 250601	India*
*C. tinctorius *L.	saff673	BJ-673	PI 193473	Ethiopia*
*C. tinctorius *L.	saff2701	BJ-2701	PI 253762	Iraq*
*C. tinctorius *L.	saff1067	BJ-1067	PI 250606	Egypt*
*C. tinctorius *L.	saffTS	TOZI SPINY	PI 271070	Sudan*
*C. curdicus *Hanelt	curd	Hanelt	W 12361	Iraq
*C. gypsicola *Iljin	gypA	UZ99a	-	Uzbekistan
*C. gypsicola *Iljin	gypB	UZ99b	-	Uzbekistan
*C. oxyacanthus *Bieb.	oxy2	K-2	PI 426428	Pakistan
*C. oxyacanthus *Bieb.	oxy1076	K-1076	PI 426185	Afghanistan
*C. oxyacanthus *Bieb.	oxy604	K-604	PI 426467	Pakistan
*C. palaestinus *Eig	palBJ	BJ-1964	PI 235663	Israel
*C. palaestinus *Eig	pal96	Ashri1917	GAT 3796	Israel
*C. palaestinus *Eig	pal97	Ashri1642	GAT 3797	Israel
*C. palaestinus *Eig	pal98	Ashri	GAT 3798	Israel
*C. persicus *Willd.	perG	Garcia-Jacas 2002	-	Turkey
*C. persicus *Willd.	per00	Aydem 157	GAT 3800	Turkey

^a ^Numbers refer to the USDA accession (PI), Vienna Museum of Natural History (W) or Gatersleben herbarium accession (GAT). No number indicates a private collection. '-' indicates missing voucher specimens. More seeds are, however, available from these collections.

^b ^Asterisks refers to the seven accessions of safflower from the seven so-called "centers of similarity" (ref. [3]; see text for details)

**Table 2:** Summary of genes surveyed and primer sequences employed.

Locus	Functional Annotation via BLAST	Primer Sequences (Forward/Reverse)
A19	At2g21330	5'-CTAGAGAACACSGARGCTAACCG
	Putative fructose bisphosphate aldolase	5'-TGGCGAAACGRGCACCYTGTTGG
A25	At2g45740	5'-TTGCATGSTCTTATCAGTCC
	Similar to putative peroxisomal membrane protein PEX11-1	5'-GAAGABCCCATCCARCAGAAGAG
A25a	-	Same as A25 Forward
		5'-TCTCTCTCATGACACCATGTAAA
A25b	-	5'-GCTCCACAGATCAGGCATTT
		Same as A25 Reverse
A27	At3g19900	5'-CTTGCAWTGAATGTCATGTGGAAG
	Unknown protein	5'-GCTCCCCARCATTTCA
A39	At2g28315	5'-ACTAGTTGGCATYTRATGGTAACA
	Putative glucose-6-phosphate/phosphate-tranlocator	5'-GCCRACAAAATTGAGCTGAAGATC
A39a	-	Same as A39 Forward
		5'-TCATGGACCAGAAATGAYGTT
A39b	-	5'-AGCACCCTTCCYTACTGCAT
		Same as A39 Reverse
B7	At4g27700	5'-AGAAAAYARCTTTGTGATTCTTGATG
	Contains Rhodanese Homology Domain	5'-GAWGARCAAGCTACTATRATCTTTG
B12	At3g55800	5'-CAAGTGGCTGCAGCCATGGG
	Sedoheptulose-bisphosphatase precursor	5'-ACATCRGGMACCATTCCWCCGGTGT
B27	At4g33250	5'-AAGGCTCTTATGGCHATGCC
	Similar to eukaryotic translation initiation factor 3 subunit 11	5'-CGGTTYTTRGCWGCTTCATCCCARAACTG
B27a	-	Same as B27 Forward
		5'-TGATGCAAAATAGTTTGTTGGAA
B27b	-	5'-TGGTGMTYCTTTTCCAATTC
		Same as B27 Reverse

Primer names followed by 'a' or 'b' were designed to amplify a given locus in two parts. Functional annotations were taken from the Genbank record for each *Arabidopsis *locus. For the unknown protein a BLASTn search failed to yield a putative function

**Table 3:** Details regarding gene regions analyzed.

Locus	Aligned Length^a^	No. Indels	N(var)^b^
A19	365	4	34
A25	486	6	42
A27	395	13	50
A39	578	4	49
B7	386	9	51
B12	408	7	57
B27	621	10	64
Combined	3239	53	267

^a ^Alignment size (bp) after removing primers, indels, and ambiguous regions

^b ^Number of variable characters

Single nucleotide polymorphisms (SNPs) were considerably more common than indels. A total of 220 SNPs were found across the full data set, resulting in an average of 1 SNP per 15 bp of sequence. Considering just the 11 safflower individuals, there were 34 SNPs, corresponding to an average of 1 SNP per 95 bp of sequence. Estimates of nucleotide diversity for *C. tinctorius*, *C. palaestinus *and *C. oxyacanthus *are presented in Table 4.

**Table 4:** Estimates of nucleotide variability and Tajima's *D*.

Locus	Taxon	θ_W_	π_Tot_	π_Sil_	Tajima's *D*^a^
A19	*C. tinctorius*	0.0041	0.0046	0.0087	0.34
	*C. palaestinus*	0.0078	0.0095	0.0180	1.03
	*C. oxyacanthus*	0.0013	0.0016	0	0.85
A25	*C. tinctorius*	0.0071	0.0102	0.0109	1.44
	*C. palaestinus*	0.0059	0.0066	0.0089	0.52
	*C. oxyacanthus*	0.0047	0.0036	0.0036	-1.30
A27	*C. tinctorius*	0.0023	0.0014	0.0006	-0.95
	*C. palaestinus*	0	0	0	n/a
	*C. oxyacanthus*	0.0063	0.0077	0.0087	1.22
A39	*C. tinctorius*	0	0	0	n/a
	*C. palaestinus*	0.0012	0.0008	0.0010	-1.05
	*C. oxyacanthus*	0.0070	0.0057	0.0084	-1.07
B7	*C. tinctorius*	0.0016	0.0010	0.0009	-0.84
	*C. palaestinus*	0.0054	0.0071	0.0112	1.43
	*C. oxyacanthus*	0.0012	0.0014	0.0025	0.85
B12	*C. tinctorius*	0.0062	0.0102	0.0175	2.20
	*C. palaestinus*	0.0067	0.0075	0.0129	0.53
	*C. oxyacanthus*	0.0277	0.0354	0.0601	1.76
B27	*C. tinctorius*	0.0020	0.0013	0.0014	-1.04
	*C. palaestinus*	0.0035	0.0044	0.0048	1.18
	*C. oxyacanthus*	0.0229	0.0184	0.0202	-1.42
Average	*C. tinctorius*	0.0033	0.0041	0.0057	0.19
	*C. palaestinus*	0.0044	0.0051	0.0081	0.61
	*C. oxyacanthus*	0.0101	0.0105	0.0148	0.13

^a ^None of the estimates of Tajima's *D *were significant

While diversity varied across loci, *C. tinctorius *generally harbored the lowest levels of diversity with Watterson's θ (θ_W_) ranging from 0 to 0.0071 (mean = 0.0033), total nucleotide diversity (π_Tot_) ranging from 0.0008 to 0.0102 (mean = 0.0041), and silent-site diversity (π_Sil_) ranging from 0.0006 to 0.0175 (mean = 0.0057). *Carthamus oxyacanthus*, on the other hand, exhibited the highest levels of diversity, with θ_W _ranging from 0.0012 to 0.0277 (mean = 0.0101), π_Tot _ranging from 0.0014 to 0.0354 (mean = 0.0105), and π_Sil _ranging from 0 to 0.0601 (mean = 0.0148). *Carthamus palaestinus *was intermediate to the other two species with θ_W _ranging from 0 to 0.0078 (mean = 0.0044), π_Tot _ranging from 0 to 0.0095 (mean = 0.0051), and π_Sil _ranging from 0 to 0.0180 (mean = 0.0081).

Because of the relatively small amount of exonic sequence included in each gene fragment (188 bp on average), individual estimates of synonymous and non-synonymous diversity must be viewed with caution. Averaging across loci, however, revealed that our estimates of non-synonymous variability are substantially lower than our estimates of synonymous variability, suggesting that diversity at these loci is primarily governed by purifying selection (data not shown). After correcting for multiple comparisons, none of the Tajima's *D *estimates were significantly different from zero (Table 4).

### Phylogenetic relationships

**Figure 1:**
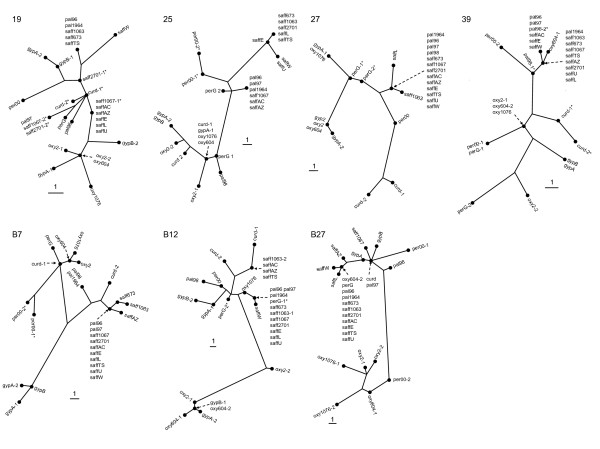
**Phylogenetic relationships among species of *Carthamus *sect. *Carthamus *based on single-gene analyses**. Neighbor-Joining trees were generated for each individual gene. Species names and accession codes are given in Table 1. Accession names followed by -1 or -2 denote alleles for a given locus. Alleles followed by a * were determined using haplotype subtraction by maximum likelihood; the remainder of the alleles were determined by cloning.

Comparison of the NJ trees produced from the single gene analyses suggests that reticulate evolution and/or incomplete lineage sorting has occurred in *Carthamus *sect. *Carthamus *(Fig. 1). While a number of overall similarities in tree topology are evident from these analyses, there are several instances in which the phylogenetic position of an individual varies depending on the gene analyzed. In some cases, individuals harbored divergent alleles at one or more loci, possibly indicating that contemporary hybridization has played an active role in the evolution of sect. *Carthamus*. Of particular note are the positions of the *C. gypsicola *alleles which are sometimes found in divergent clades (e.g., for genes A19 and B12). Some *C. oxyacanthus *alleles show a similar pattern (e.g., genes A39 and B27). When comparing the seven trees for the individual loci, however, some patterns begin to emerge. Overall, *C. oxyacanthus *is most often found to be relatively distantly related to *C. tinctorius*, and frequently associated with alleles from *C. gypsicola*. Of particular note is the close relationship between individuals of *C. tinctorius *and *C. palaestinus*, suggesting that the species most closely related to safflower (and hence its most likely progenitor) is *C. palaestinus *(Fig. 1).

**Figure 2:**
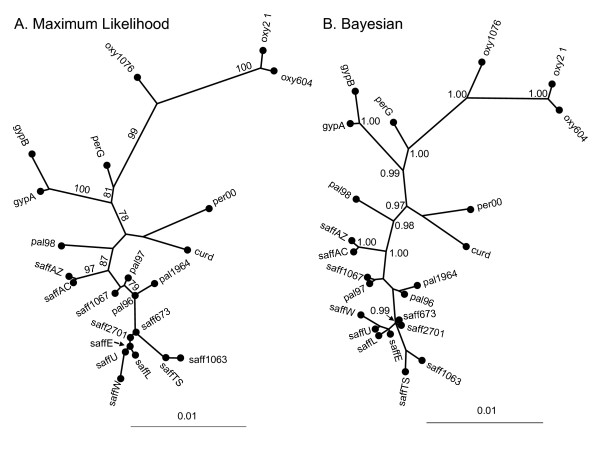
**Phylogenetic relationships among species of *Carthamus *sect. *Carthamus *based on a combined analysis of seven nuclear genes**. Maximum likelihood (A) and Bayesian (B) trees generated for the combined dataset. Bootstrap values (> 75%) for the ML tree and posterior probabilities (> 0.90) for the Bayesian tree are given alongside branches. Species names and accession codes are given in Table 1.

Despite the occurrence of some incongruities between loci, the NJ, ML and Bayesian trees based on the combined data (which are nearly identical to each other in topology; Fig. 2 and data not shown) are in overall agreement with our interpretations of the single gene analyses. *Carthamus oxyacanthus *is resolved as the species most distantly related to safflower, with high ML bootstrap and Bayesian posterior probabilities. Within *C. oxyacanthus *the two lines from Pakistan are more closely related to each other than they are to the line from Afghanistan. *Carthamus persicus *appears to be paraphyletic, perhaps due to recent gene flow. As predicted from the individual gene trees, *C. palaestinus *is the most closely related species to safflower, and we conclude that this species is the most likely progenitor of safflower. In the Bayesian analysis, all four *C. palaestinus *individuals are found in a well-supported clade along with all of the safflower individuals. Similarly, in the ML analysis, three of the four individuals of *C. palaestinus *are found in such a clade (87% BS), with the fourth resolving at the base of this clade along with individuals of *C. curdicus *and *C. persicus*. Some relationships can also be resolved among safflower individuals; for example, saffAZ and saffAC form a well-supported clade at the base of the safflower/*C. palaestinus *group. Relationships between the other cultivars are, however, poorly-supported.

## Discussion

### Origin of safflower

A close relationship between members of sect. *Carthamus *has been proposed based on data from crossing studies [reviewed in [12,13]], the identification of natural hybrids amongst some species within the section [1,14], and phylogenetic analyses involving some members of the section [9]. Prior to the present investigation, however, the phylogenetic relationships amongst all species within sect. *Carthamus *had not been investigated, and the identity of the progenitor of safflower had only been hypothesized. While Ashri & Knowles [14] proposed that safflower was derived from hybridization between *C. oxyacanthus *and *C. persicus*, this hypothesis is clearly not supported by our results. Rather, *Carthamus palaestinus *and safflower are found in the same clade indicating a close relationship between these species. We thus propose that *C. palaestinus*, which is native to the deserts of southern Israel and western Iraq, is the wild progenitor of safflower. Safflower and *C. palaestinus *share a self-compatible breeding system [12]; thus, the near absence of heterozygous loci in these species (Fig. 1) is unsurprising. *Carthamus persicus *and some populations of *C. oxyacanthus *are self-incompatible, and this is evident from the presence of much more heterozygosity in these species (Fig. 1). The cause of the non-monophyly of *C. persicus *remains unknown due to the small sample sizes necessarily employed here; however the retention of ancestral polymorphism and/or contemporary gene flow (*C. persicus *is self-incompatible) could be responsible.

As noted in the Introduction, Knowles [3] recognized seven distinct "centers of similarity" of safflower, including the Far East, India-Pakistan, the Middle East, Egypt, Sudan, Ethiopia and Europe. Interestingly, our data provide little support for the distinctiveness of safflower accessions from these disparate geographic locales. Indeed, while there is a small amount of phylogenetic structure apparent within safflower, it appears that most of the accessions surveyed herein are highly similar at a genetic level. Moreover, much of the substructuring within safflower is not well-supported in either the Bayesian or ML analyses. The exceptions to this are a pair of accessions from the USA and Canada (saffAZ and saffAC, respectively), and possibly one accession from Egypt (saff1067). Considering what we know about the history of safflower cultivation, the North American accessions (saffAZ, saffAC, and saffU) are presumably recent introductions, and from our data it appears that they may be derived from relatively divergent ancestral stocks (Fig. 2). Further investigation of the 'seven centers' hypothesis, will require the development and application of more variable markers to a much more robust sampling of the available safflower germplasm. While a recent investigation of safflower cultivars using RAPDs, ISSRs and AFLPs revealed some genetic structuring within the species [10], the authors did not address the question of whether or not the seven morphological centers of diversity correspond to genetic subgroups within safflower.

### Levels of nucleotide diversity

The domestication of plant species is typically accompanied by a reduction in genetic diversity resulting from the population genetic bottleneck that occurs during domestication. Although results vary across species, crops generally harbor ca. two-thirds of the diversity that is present in their wild progenitors [15]. We found a similar value here, with a 20–30% (depending on the measure) reduction in nucleotide diversity in safflower as compared to *C. palaestinus *(Table 4). Because θ_W _is roughly proportional to heterozygosity, we can further conclude that a randomly selected pair of safflower sequences will differ at an average of 1 out of every 303 bp (i.e., 1/0.0033 ≅ 303). This makes safflower considerably less diverse than crops such as maize (1 out of every 105 bp [16]) and sunflower (1 out of every 140 bp [17]) but far more diverse than crops such as soybean (1 out of every 1030 bp [18]).

## Conclusion

Insights into the origin of crop plants and knowledge of the identities of their progenitors are of great value in both basic and applied research programs. For example, the comparative analysis of crop plants and their wild progenitors can shed light on the genetic mechanisms underlying organismal evolution [19,20]. Similarly, comparative analyses of this sort can be a powerful tool for identifying genes underlying agronomically-important traits [21-23]. The identification of *C. palaestinus *as the wild progenitor of safflower opens the door for such analyses within the genus *Carthamus*. Moreover, because safflower is a modern-day oilseed crop and a member of the same family as cultivated sunflower, which has been the subject of a great deal of recent study [24,25], the initiation of such work in safflower would create a comparative framework for studying the evolution of oilseed crops within the Asteraceae.

## Methods

### Plant materials and DNA extraction

Tissue for DNA extraction was either obtained from live plants grown from seed or from herbarium specimens (Table 1). Seeds were obtained from archived collections held at the USDA Western Regional Plant Introduction Station in Pullman, WA. These included 11 accessions of *C. tinctorius *L., three accessions of *C. oxyacanthus *and one accession of *C. palaestinus*. In addition Dr. R. Vilatersana (Institut Botànic de Barcelona) kindly provided seeds of *C. gypsicola *and *C. persicus*. Herbarium specimens of *C. palaestinus *(three accessions) and *C. persicus *(one accession) were provided by the IPK Gatersleben Herbarium (GAT), and *C. curdicus *(one accession) was provided by the Vienna Museum of Natural History (Herbarium W). All species are presumed to be diploid based on prior investigations [reviewed in [11]].

For the live plants, seeds were clipped with a razor blade and germinated on damp filter paper in Petri dishes (48 hours dark/48 hours light). Seedlings were then planted in soil and grown in the greenhouse. Total genomic DNA was then isolated from 100 mg of leaf tissue using the DNeasy plant mini kit (Qiagen, Valencia, CA). For the herbarium extractions, tissue lysis and extraction followed the same protocol as the fresh leaf tissue except that only ca. 20 mg of leaf tissue was used.

### Locus selection and sequencing

The seven nuclear genes used in this study (Table 2) were selected from a set of universal markers that were recently developed for use in the Asteraceae [26]. The loci selected for inclusion in this study all produced a single amplicon that could be sequenced directly (only those individuals that were heterozygous for insertions/deletions were cloned). Three of the loci (A25, A39, and B27; Table 2) did not amplify well in the herbarium material, presumably due to DNA degradation; internal primers were thus designed to amplify these loci in two overlapping segments that were later aligned into a single contig. For A39 the first portion still could not be amplified in the herbarium specimens, and hence was scored as missing data for those individuals.

PCR was performed in a 20 μl total volume containing 20 ng of template DNA, 30 mM Tricine pH 8.4-KOH, 50 mM KCl, 2 mM MgCl_2_, 100 μM of each dNTP, 0.2 μM of each primer, and 2 units of *Taq *polymerase. Thermal cycling followed a 'touchdown' protocol, with a final annealing temperature of 50° or 55°C, as follows: (1) initial denaturing step of 3 minutes at 95°C, (2) ten cycles of 30 s denaturation at 94°C, 30 s annealing at 60° or 65°C (annealing temperature was reduced by one degree per cycle), 45 s extension time at 72°C, (3) 30 cycles of 30 s at 94°C, 30 s at 50° or 55°C, 45 s at 72°C, and (4) a final extension of 20 m at 72°C. Following PCR amplification, the presence of amplicons was confirmed via agarose gel electrophoresis.

To prepare for DNA sequencing, 10 μl of each PCR product was incubated at 37°C for 45 m with 4 units of Exonuclease I and 0.8 units of Shrimp Alkaline Phosphatase (USB, Cleveland, OH). Enzymes were subsequently denatured by heating to 80°C for 15 minutes. Purified PCR amplicons (0.5 – 2 μl depending on approximate concentration) were then sequenced with the primers used for the initial PCR. DyeNamic (Amersham, Piscataway, NJ) or BigDye v3.1 (Applied Biosystems, Foster City, CA) chemistry was used for the sequencing following the manufacturers' protocols with minor modifications. Unincorporated dyes were removed from the sequencing reactions via Sephadex (Amersham) clean-up and sequences were resolved on a Basestation (MJ Research, San Francisco, CA) or ABI 3730xl (Applied Biosystems) automated DNA sequencer. For individuals that were heterozygous for indels at a particular locus (as evidenced by the initial sequencing chromatogram), the unpurified PCR product was cloned using the pDrive (Qiagen) or TOPO TA (Invitrogen, Carlsbad, CA) cloning vectors following the manufacturers' protocols. In order to protect against *Taq *errors, PCR products from five positive clones per cloning reaction were then prepared and sequenced as above, except that the T7 and M13 universal vector primers were used.

### Data analyses

DNA sequences were edited using Chromas 2.12 (Technelysium, Helensvale, Australia). Heterozygous bases from uncloned PCR products were detected by the presence of double peaks and coded following the conventions of the International Union of Biochemistry and Molecular Biology. From these, haplotypes were resolved using the maximum likelihood algorithm PL-EM [27] within the HapAnalyzer software [28]. Sequences were aligned using Clustal W2 [29] with the default settings, followed by manual adjustments. Indels were scored as additional characters using GapCoder [30], although regions that could not be aligned unambiguously and length variants at simple-sequence repeats were excluded from the analysis. Heterozygotes were common, and alleles were kept separate for the individual gene phylogenetic analyses. For the combined dataset, however, the phase of alleles across loci could not be reliably determined. As such, pairs of cloned alleles were collapsed into a single genotype for each gene and then the seven genes were concatenated for each individual.

Estimates of nucleotide diversity (π and θ, calculated on a per-site basis) as well as Tajima's *D *[31] were obtained for the three taxa with three or more samples (*C. tinctorius*, *C. palaestinus*, and *C. oxyacanthus*) using the software package DnaSP 4.00.5 [32,33]. Neighbor-Joining trees were generated separately for each locus and for the combined dataset using PAUP* ver. 4.0b [34]. The combined dataset was also subjected to Maximum likelihood (ML) and Bayesian analyses. ML analysis was carried out using PHYML v2.4.4 [35] under the HKY+Γ model of molecular evolution with four substitution rate classes with 500 bootstrap replicates. Bayesian analysis was conducted with MrBayes [36] as implemented in the Geneious package (v3.0.6; Biomatters Ltd., Auckland, New Zealand). MCMC analysis was run with four chains simultaneously for 1,100,000 generations, subsampling every 200 generations. Samples prior to the generation 100,000 were treated as burn-in and discarded.

## Authors' contributions

MAC and JMB conceived the investigation, carried out the analyses and wrote the paper. MAC carried out the PCR and sequencing. All authors read and approved the final manuscript.
